# *sc*BrainMap: a landscape for cell types and associated genetic markers in the brain

**DOI:** 10.1093/database/baad035

**Published:** 2023-05-17

**Authors:** Yuhao Chi, Ruicheng Qi, Yue Zhou, Huige Tong, Hanbo Jin, Christoph W Turck, Wei-Hua Chen, Guang-Zhong Wang

**Affiliations:** CAS Key Laboratory of Computational Biology, Shanghai Institute of Nutrition and Health, University of Chinese Academy of Sciences, Chinese Academy of Sciences, No. 320 Yueyang Road, Shanghai 200031, China; CAS Key Laboratory of Computational Biology, Shanghai Institute of Nutrition and Health, University of Chinese Academy of Sciences, Chinese Academy of Sciences, No. 320 Yueyang Road, Shanghai 200031, China; Department of Mathematics, Guangxi University, No. 100 East University Road, Nanning, Guangxi 530004, China; CAS Key Laboratory of Computational Biology, Shanghai Institute of Nutrition and Health, University of Chinese Academy of Sciences, Chinese Academy of Sciences, No. 320 Yueyang Road, Shanghai 200031, China; Department of Bioinformatics and Systems Biology, Key Laboratory of Molecular Biophysics of the Ministry of Education, Hubei Key Laboratory of Bioinformatics and Molecular-Imaging, Center for Artificial Intelligence Biology, College of Life Science and Technology, Huazhong University of Science and Technology,No. 1037 Luoyu Road, Wuhan 430074,China; Proteomics and Biomarkers, Max Planck Institute of Psychiatry, No. 2-10 Kraepelinstr, Munich 80804, Germany; Department of Bioinformatics and Systems Biology, Key Laboratory of Molecular Biophysics of the Ministry of Education, Hubei Key Laboratory of Bioinformatics and Molecular-Imaging, Center for Artificial Intelligence Biology, College of Life Science and Technology, Huazhong University of Science and Technology,No. 1037 Luoyu Road, Wuhan 430074,China; CAS Key Laboratory of Computational Biology, Shanghai Institute of Nutrition and Health, University of Chinese Academy of Sciences, Chinese Academy of Sciences, No. 320 Yueyang Road, Shanghai 200031, China

## Abstract

The great variety of brain cell types is a fundamental element for neuronal circuits. One major goal of modern neuroscience is to decipher the various types of cellular composition and characterize their properties. Due to the high heterogeneity of neuronal cells, until recently, it was not possible to group brain cell types at high resolution. Thanks to the single-cell transcriptome technology, a dedicated database of brain cell types across species has been established. Here, we developed *sc*BrainMap, a database for brain cell types and associated genetic markers for several species. The current *sc*BrainMap database contains 4881 cell types with 26 044 genetic markers identified from 6 577 222 single cells, which link to 14 species, 124 brain regions and 20 different disease states. *sc*BrainMap enables users to perform customized, cross-linked, biologically relevant queries for different cell types of interest. This quantitative information facilitates exploratory research on the role of cell types with regard to brain function in health and disease.

**Database URL** https://scbrainmap.sysneuro.net/

## Introduction

Defining the complete collection of brain cell types is of great importance to the field of neuroscience and has recently become a hot research topic, as it provides the basis for understanding the cellular diversity of brain circuits and networks (1–9). By combining multi-omics datasets, sequence information for >500 000 cells from mouse primary motor cortex was obtained, resulting in the discovery of 56 inhibitory and excitatory neuron cell types (2). Using multiplexed error-robust fluorescence *in situ* hybridization, a total of 95 cell clusters with differential spatial organization were identified in different layers of this brain region (4). These neuronal and non-neuronal cell clusters are largely conserved across mammalian species (3). These datasets hold immense value not only in revealing the evolutionary dynamics of the brain at single-cell level (10) but also in dissecting detailed patterns at the tissue level (11–15). However, a comprehensive brain cell-type resource covering different species and brain regions is still lacking.

The development of an integrated resource for molecular classification of cell types is not trivial. Many factors may affect the cellular composition of the tissues collected, including the type of brain region (16), species (3), developmental stage (17) and disease state (18). Single-cell RNA sequencing (scRNA-seq) technology enables the clustering of the transcriptome of individual cells and is therefore well suited to address the complexity and dynamics posed by the diverse cell types of the nervous system (19). However, because a rigorous definition of cell types is not yet available, identifying and designating the cellular clusters of the brain is a difficult task (19). Additionally, the designation of many neuronal cell types, such as chandelier cells and pyramidal cells, is either based on their morphology and anatomical location in the brain or based on their electrophysiological properties (20). In general, the number of cell types mapped by single-cell transcriptomics is much larger than the one identified based on conventional techniques. How to best integrate the multimodal information on brain cell types is a difficult problem because distinct cell types defined by their single-cell transcriptome may have similar morphological and electrophysiological characteristics (8).

Currently available brain cell atlas (21, 22), including the Allen Brain Atlas cell types database (https://celltypes.brain-map.org/), has only data for the human or the mouse brain. With the advent of single-cell sequencing technologies, datasets for other well-studied organisms, such as *Drosophila melanogaster* and *Danio rerio*, have been accumulated (23, 24). So far, none of the public databases have data for non-model species, such as reptiles (25). Including these phylogenetically diverse species will facilitate research across species, which is essential for the investigation of cell-type evolution. Thus, there are an urgent need and an unprecedented opportunity for a comprehensive data collection on brain cell types including multiple species and conditions.

In this work, we present *sc*BrainMap, a brain cell-type database across multiple evolutionary-relevant species. This database was developed based on the manually curated 715 single-cell transcriptome datasets. A total of 4881 brain cell types have been annotated with 26 044 marker genes, covering 124 brain regions of 14 species. By querying *sc*BrainMap, users can conveniently identify a specific cell type that exists in a particular brain region. The expression profile of each genetic marker can then be visualized and downloaded for further analysis. Distinct developmental periods and disease states are also present in *sc*BrainMap. This database represents a reference catalog of brain cell types and genetic markers and provides insights into the function and cellular composition of different brain circuits across evolution.

## Material and methods

### Single-cell data collection and curation

**Figure 1.**
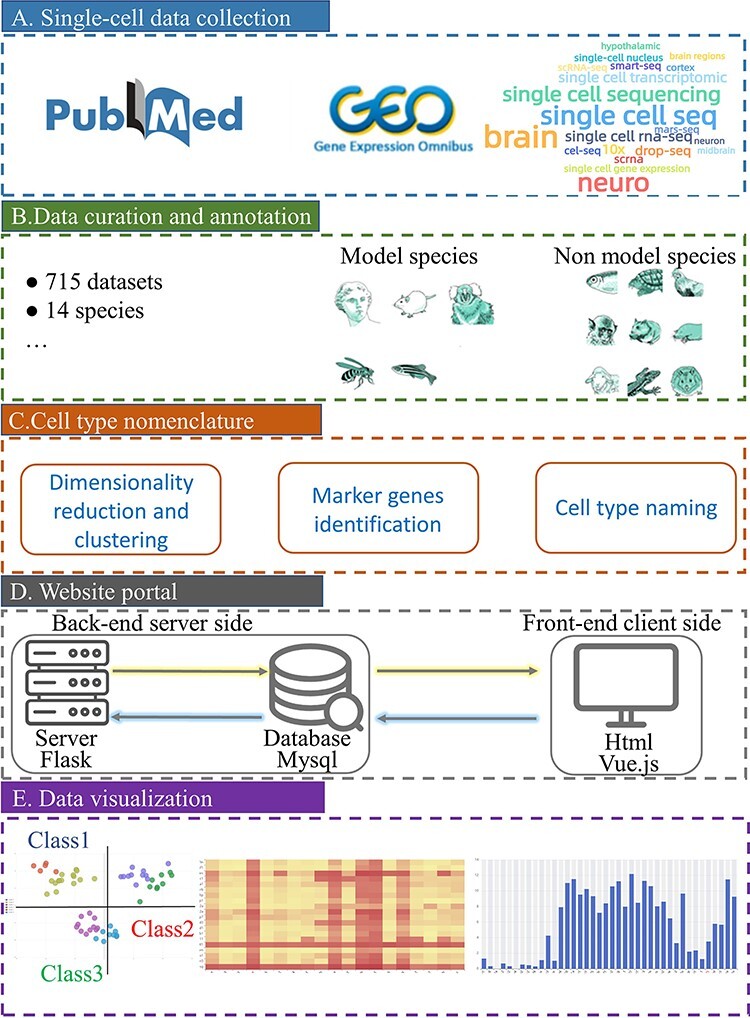
Schematic overview of *sc*BrainMap database construction. (A) Literature search in PubMed and GEO with a list of well-defined keywords. (B) All datasets were manually curated for various species. (C) Cell-type clustering and naming were performed. (D) The database portal was built for both the front end and the back-end. (E) Convenient visual and interactive user interfaces were implemented.

To obtain a comprehensive literature collection on brain single-cell sequencing data, we searched PubMed database and Gene Expression Omnibus (GEO) (26) from 2015 with a list of keywords in the title and abstract, utilizing the R package RISmed (Figure 1A). The keywords used are all related to the single-cell transcriptome: ‘single cell seq’, ‘single cell sequencing’, ‘single cell rna-seq’, ‘single cell transcriptomic’, ‘10x, drop-seq’, ‘scrna’, ‘smart-seq’, ‘cel-seq’, ‘mars-seq’, ‘single-cell nucleus’, ‘single cell gene expression’ and ‘scRNA-seq’. We then combined brain-related keywords in the subsequent filtering: ‘brain’, ‘neuro’, ‘cortex’, ‘hypothalamic’, ‘neuron’, ‘midbrain’ and ‘brain regions’. We identified 2151 potential articles through this automatic text-mining approach. We manually screened all 2151 articles to identify those related to single-cell transcriptome data in the brain. We then eliminated articles where transcriptome expression data were unavailable due to patient privacy or the datasets that contain insufficient cells. In total, 210 articles were retained for our database, with some of them containing multiple datasets. Our final dataset includes 715 single-cell transcriptome datasets from the brain, which can be searched and compared using our database (Figure 1B).

### Cell-type annotation strategy

We annotated and designated each brain cell cluster computationally (Figure 1C). First, single-cell transcriptome data were clustered iteratively by using scrattch.hicat (16), which is based on principal component analysis dimension reduction and cluster merging process. Second, for each species, a high-quality dataset was established as a reference dataset (Supplementary Table S1). All major cell classes (e.g. GABAergic, glutamatergic and non-neuron) and subclasses (e.g. Lamp5, L5) of the dataset to be annotated were mapped to the reference data by using Seurat’s TransferData method. We carefully selected a reference dataset for each species. For human, mouse and monkey, we chose datasets sampled from the primary motor cortex (3) as the reference dataset (Supplementary Table S1), as these species have a well-conserved hierarchical organization of cell types. Additionally, these datasets are well annotated. For other species, we selected data based on the three-tiered structural annotation of cell class, subclass and cell type, as this approach provides the most comprehensive classification of cell types (24, 25, 27, 28). At the same time, differentially expressed genes (DEGs) for each cell cluster were calculated by receiver operating characteristic method of Seurat’s FindAllMarkers (29). The top 20 DEGs derived for each cell cluster were selected, and the DEGs with high cell-type specific transcriptional signatures were stored for further usage. Third, the top three genes with the highest expression specificity among these stored DEGs were regarded as marker genes for the given cell type. If no DEGs were detected for a cell type, an in-house random forest algorithm was performed to identify potential marker genes. Finally, brain cell-type designation was conducted by combining Seurat-supervised classifier classification of cell classes and the top marker gene identified, following a strategy proposed previously (30).

### Development of *sc*BrainMap

Open-sourced software packages were used to implement the whole database, including user-friendly web interfaces (Figure 1D). The computational process for cell-type identification from single-cell data was run in R (4.0.3), utilizing the Seurat (4.0.1) and scrattch.hicat (1.0.0) packages. The in-house random forest algorithm was developed in Python (3.7.3) using the Scikit-Learn (0.23.1) package. *sc*BrainMap database was deployed on the Apache (2.4.37) server of Centos Linux. All data are stored in MySQL (8.0.0) database. The web interface was constructed using Vue.js (2.9.6) as a JavaScript framework combined with dynamic HyperText Markup Language pages (Figure 1E). To transfer back-end data to the front end, we introduced libraries in Vue.js including eCharts (5.3.3), Element-UI (2.15.9), The bootstrap (5.1.3), SASS (1.13.4) and Flask (2.1.1) framework in Python (3.7.3).

## Results

### Overview of *sc*BrainMap

**Figure 2.**
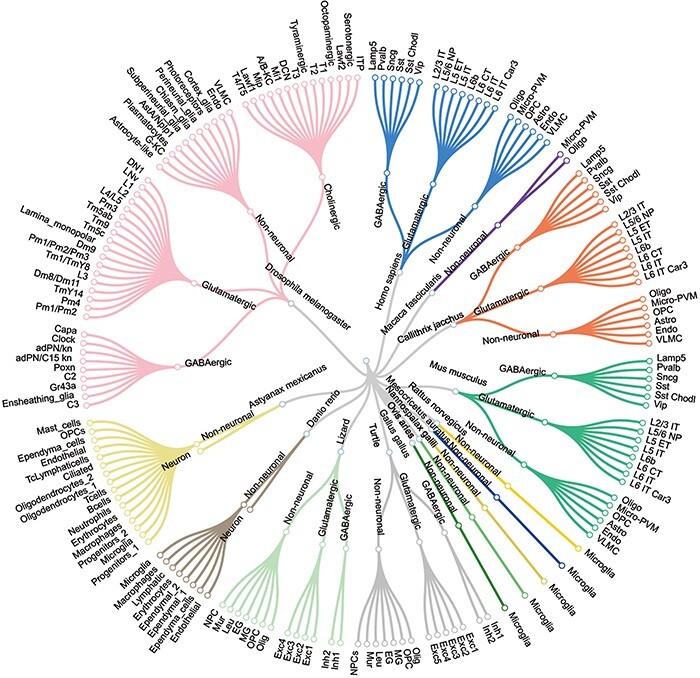
The major cell types and sub-cell types identified for each species.

*sc*BrainMap can be freely accessed at https://scbrainmap.sysneuro.net/. *sc*BrainMap database currently includes 715 brain single-cell transcriptome datasets derived from 210 articles, containing 6 577 222 cells, 4881 cell types and 26 044 marker genes. The whole data collection covers 14 species (Figure 2), including 5 model species and 9 non-model species. The model species we collected are *Homo sapiens, Mus musculus, Callithrix jacchus*, *D. melanogaster* and *D. rerio*, while the non-model species included are *Astyanax mexicanus, Chelydra serpentina, Gallus gallus, Macaca fascicularis, Mesocricetus auratus, Nannospalax galili, Ovis aries, Podarcis muralis* and *Rattus norvegicus*. The top five species that contain the largest number of cell types are listed in Table 1, with detailed information of marker genes and single cells incorporated. For instance, the largest number of single-cell datasets in the database is from mouse, with 3 916 903 single cells and 10 525 marker genes.

**Table 1.** Statistics on brain cell types for the top five species

Species	Cell-type number	Number of single cells	Number of marker genes
*Homo sapiens*	1931	2 275 378	11 582
*Callithrix jacchus*	354	119 366	2 058
*Mus musculus*	1680	3 916 903	10 525
*Danio rerio*	265	57 600	1 476
*Astyanax mexicanus*	189	56 687	1 163

In addition, we found that these datasets are associated with 124 brain regions, 128 developmental stages and 20 different disease states, most of which based on samples from either mouse or human. For mouse, we collected 86 different brain regions and 91 different developmental stages together with 13 different disease states. For human, 56 different brain regions, 33 different developmental stages and 12 different disease states including coronavirus disease were registered.

### *sc*BrainMap search options

*sc*BrainMap provides very convenient search functions. There are several ways performing the search function. The users can search by:

- Gene symbol: gene symbol can be searched in a fuzzy function, with three letters to display the candidate items automatically. In addition to the candidate gene name, the results also display which species the gene belongs to.
- Cell type: a specific cell type of interest to the user can be searched by the name of cell type. The results also display which species that cell type is derived from. If the user is uncertain about the cell type he or she is looking for, a webpage containing all the cell types reconstructed for a given species is provided.
- Species name: search with species name (e.g. mouse) was implemented.
- Brain region: an autosuggest search box is available for brain regional search. The users can also browse all the brain regions for a species to examine a potential brain region of interest. Currently, this function is available for three model species: human, monkey and mouse.
- Developmental period: search for a specific developmental period is implemented for 10 of the 14 species, i.e. *H. sapiens*, *M. musculus*, *C. jacchus*, *D. melanogaster*, *G. gallus*, *M. fascicularis*, *M. auratus*, *N. galili*, *O. aries* and *R. norvegicus*.
- Disease state: samples from brain disease patients can also be searched and browsed, such as Alzheimer’s disease (AD), Huntington’s disease and major depressive disorder.

A batch mode search option is also available. Users can download all the annotated terms of each dataset and further customize their search by using keywords like ‘Species’, ‘Brain Region’, ‘Seq method’. This function is particularly useful for bioinformaticians who have interest in further data mining.

### Database web implementation

**Figure 3.**
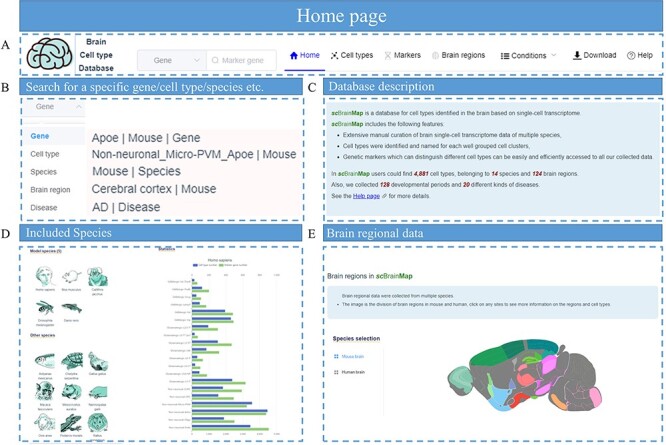
Screenshot of *sc*BrainMap database homepage. (A) The navigation bar. (B) Live search box. (C) Database introduction. (D) Statistical plot for each species and (E) brain regions.

*sc*BrainMap provides a concise interactive web page with several pages including ‘Home’, ‘Cell Types’, ‘Makers’, ‘Brain Regions’, ‘Conditions’, ‘Download’ and ‘Help’ in the navigation bar (Figure 3A). Users can search the entire database conveniently through the live search box in the top panel of the home page (Figure 3B). This page also shows statistical summaries for the datasets covered in the database (Figure 3C). The species of interest can be selected, and the overall statistical analysis including the number of cell types and marker genes of the major cell and sub-cell classes of the species can be viewed (Figure 3D). The brain region of interest can also be filtered, which will then show the cell types identified in the selected brain region (Figure 3E).

**Figure 4.**
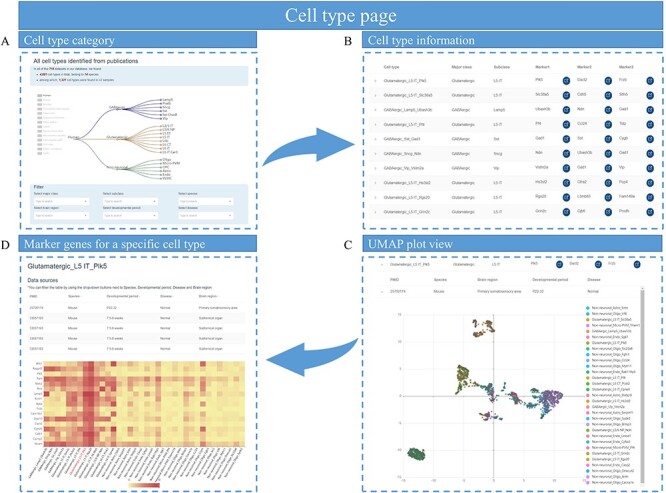
Cell types page of *sc*BrainMap. (A) At the top of the page is a tree of major class and subclass cell types for each species. (B) The cell-type information table contains the name of the cell type, major class, cell subclass and their top marker genes. (C) An UMPA plot of single-cell clustering is displayed. (D) Heatmaps of DEG in cell types are visualized.

On the ‘Cell types’ page, clicking on a species will show the visualization of the major classes and subclasses for distinct cell types in this species (Figure 4A). Selecting a particular major cell class or subclass will display all the cell types that belong to the current category (Figure 4B). If the drop-down box for a particular cell type is selected, the dataset from which the current cell type was derived is displayed along with a Uniform Manifold Approximation and Projection (UMAP) plot of all single cells in that dataset (Figure 4C). Clicking on the name of the current cell type takes the user to a page with more detailed information, such as in which dataset the cell type has been identified, and a heatmap of the expression patterns of the top DEGs for users to explore (Figure 4D).

**Figure 5.**
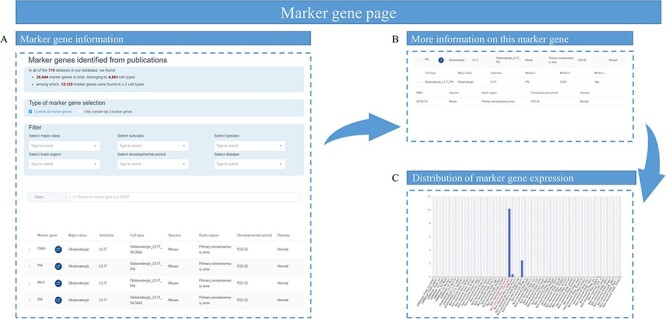
Overview of marker gene page. (A) The marker gene information table containing marker gene name, the link to National Center for Biotechnology Information website and the cell types that contain this marker gene. (B) Sample information of this marker gene, such as the species, brain region, developmental stage and disease status. (C) A bar graph showing the expression of a marker gene among different cell types.

On the generated ‘Markers’ page, the user can search for the target gene of interest. Specific conditions of major cell class, subclasses, species, brain regions, developmental stages and diseases can be filtered for marker genes (Figure 5A). Selecting a specific gene drop-down box shows which cell type contains this highly expressed gene (Figure 5B). Clicking this gene will jump to the marker gene details page, with a bar graph of the gene expression distribution in the current dataset at the bottom (Figure 5C).

On the ‘Brain regions’ page (Figure S1A), the users can select the species of interest and filter the genetic markers for different brain regions. They can also click on different brain regions to screen all of them for different cell types. Alternatively, the user can select other ‘Conditions’, such as ‘Developmental stage’ (Figure S1B) or ‘Disease’ (Figure S1C), to look for genetic markers of interest. Then, a manually annotated copy of each article and a link to the annotation results can be found on the ‘Download’ page (Figure S1D). Details of the data (such as sequencing methods and platforms) can also be found there. Finally, a documentation page (Help page) is also available to assist the user. Contact information is provided to welcome any suggestions, comments or questions related to the *sc*BrainMap database.

### Application example 1: exploring dopamine receptor D2–related cell types

**Figure 6.**
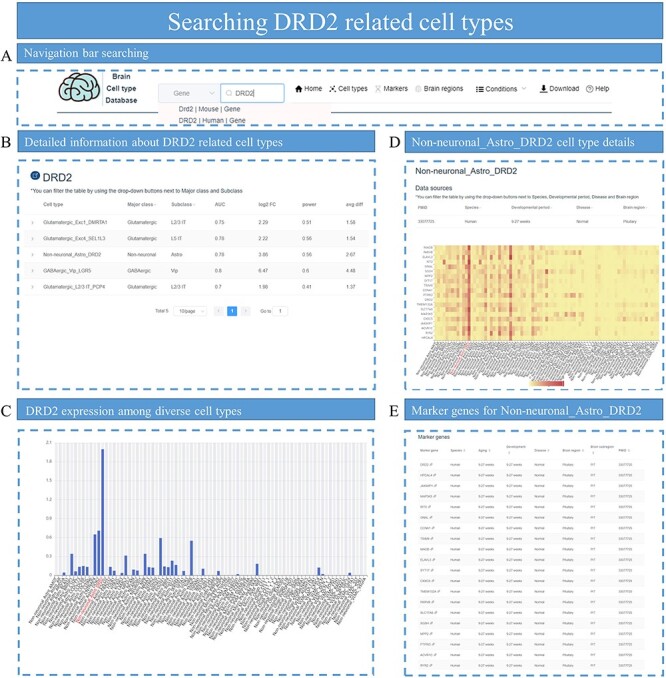
Identifying *DRD2*-related cell types. (A) Search for *DRD2* in the search box of the homepage. (B) All cell types related to *DRD2* are displayed, with parameters associated with DEGs such as log2 FC and power value. (C) Bar plot shows the distribution of *DRD2* expression among cell types in that specific dataset. (D) The heatmap plot for top DEG expression distribution is displayed for cell-type Non-neuronal_Astro_DRD2. (E) All the top marker genes for cell-type Non-neuronal_Astro_DRD2 are provided.

Dopamine receptor D2 (*DRD2*) is a gene that regulates synthesis, storage and release of dopamine, which can increase the risk of neuropsychiatric disorders if mutated (31). In this example, we tried to explore *DRD2*-related cell types. First, we searched for ‘DRD2’ in the live search box in the navigation bar of *sc*BrainMap home page (Figure 6A). Selecting this gene to jump to the detailed results page of *DRD2* (Figure 6B) shows in which cell-type *DRD2* is a significant DEG. We found that *DRD2* exists in five cell types and is identified under different conditions. We further screened these cell types with the result that Non-neuronal_Astro_DRD2 is the cell type in which *DRD2* is specifically expressed as a marker gene that is located in pituitary. Selecting this cell type reveals the expression pattern of *DRD2* in the dataset (Figure 6C). Indeed, it shows that *DRD2* is specifically highly expressed in Non-neuronal_Astro_DRD2 cell type. The cell-type page search shows that Non-neuronal_Astro_DRD2 is identified in one dataset. The expression patterns of the top DEGs belonging to Non-neuronal_Astro_DRD2 among all cell types are also visualized (Figure 6D). With a similar strategy, the cell types related to other well-studied genes such as *PER1*, *PER2*, *FOXP2*, *Vip* and *Syt6* were also explored (Supplemental Figures S2–6).

### Application example 2: exploring markers for Glutamatergic_L5 IT_Rspo1

**Figure 7.**
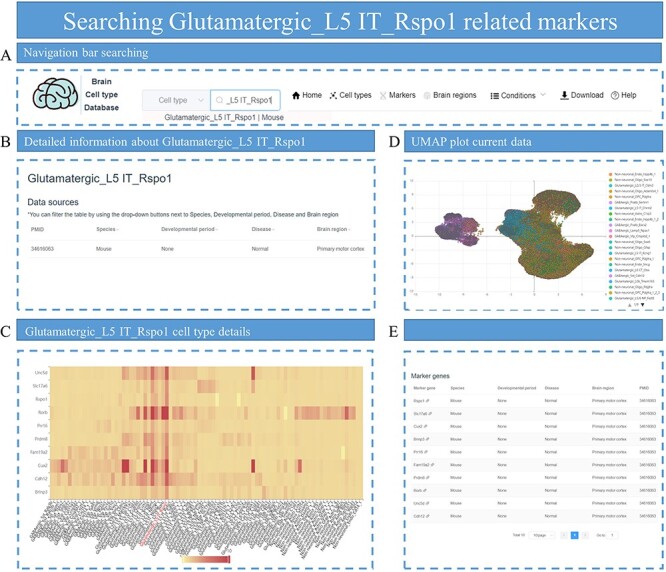
The process of searching for markers related to Glutamatergic_L5 IT_Rspo1. (A) Enter ‘Glutamatergic_L5 IT_Rspo1’ in the search box on the homepage. (B) The data related to Glutamatergic_L5 IT_Rspo1 are displayed, including parameters such as PMID, species and other conditions. (C) A heatmap displays the distribution of marker genes for Glutamatergic_L5 IT_Rspo1 among different cell types in the specific dataset. (D) The UMAP plot shows all cells in the dataset and provides annotation information on cell types. (E) All marker genes for the Glutamatergic_L5 IT_Rspo1 cell type are listed.

R-spondin1 (*Rspo1*) is a specific marker for L4/5 IT neurons (32). In this example, we tried to find marker genes for Glutamatergic_L5 IT_Rspo1 neurons. First, we select the ‘cell type’ option in the search box and enter Glutamatergic_L5 IT_Rspo1 to search (Figure 7A). The search box will jump to the cell-type details page, showing all the data identified for the Glutamatergic_L5 IT_Rspo1 cell type (Figure 7B). Selecting a specific set of data will reveal all cell types included in the set of data, as well as the expression patterns of marker genes identified by Glutamatergic_L5 IT_Rspo1 in all cell types (Figure 7C). Searching PubMed Identifier (PMID) of this set of data can also obtain UMAP of all cells contained in this set of data and annotation information of cell types (Figure 7D). All the marker genes identified in Glutamatergic_L5 IT_Rspo1 cell types will also be displayed on the cell-type details page (Figure 7E).

### Application example 3: exploring cell types and markers for patients with AD

**Figure 8.**
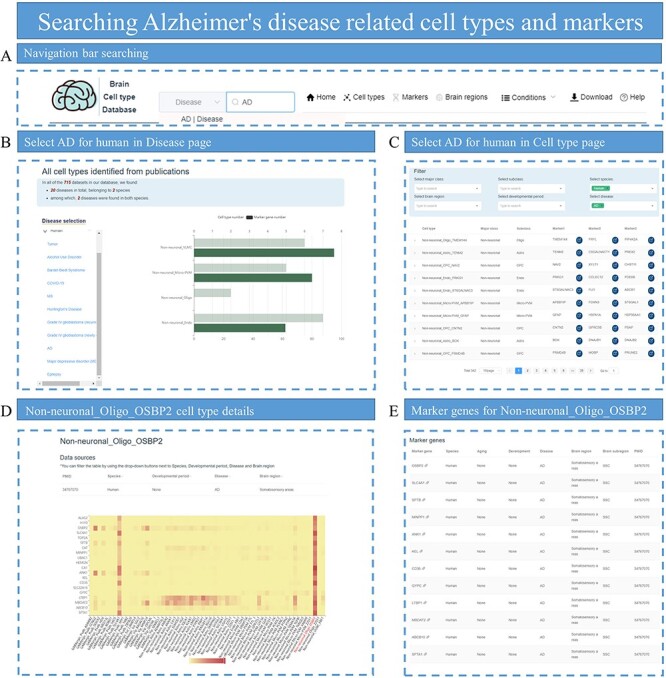
Searching for AD-related cell types and markers. (A) Enter ‘Alzheimer’s disease’ in the search box of the homepage. (B) Select AD on the Disease page to view related cell types, which are displayed with the number of identified marker genes. (C) Select species, disease and subclass conditions on the Cell type page to display all eligible cell types. (D) Display the heatmap plot for the expression distribution of top DEGs in cell-type Non-neuronal_Oligo_OSBP2. (E) Provide all marker genes for cell-type Non-neuronal_Oligo_OSBP2.

Alzheimer’s disease (AD) is a neurodegenerative disease that causes memory impairment and cognitive decline. Glial cells, including astrocytes, microglia and oligodendrocytes, have been implicated in the pathogenesis of AD (33). We can look at the cell types associated with AD on both pages. First of all, we can search the Disease page to select human AD. The number of cell types identified under current conditions and the number of marker genes identified by corresponding cell types will be displayed on the right side (Figure 8B). At the same time, data can be screened from the Cell type page, and all relevant cell types can be displayed by selecting species, diseases and cell subclass (Figure 8C). To explore the Oligo subclass, we can choose the cell types whose subclass is Oligo, such as Non-neuronal_Oligo_OSBP2. Click on this cell type to display the expression distribution of marker for the current cell type in all cell types (Figure 8D), as well as the detailed information of all marker genes identified (Figure 8E).

## Discussion

*sc*BrainMap is the most comprehensive collection of brain cell types and their associated genetic markers to date. It provides a convenient tool for searching for specific cell types or genetic markers in a given brain region. Our customized pipeline for cell-type clustering and nomenclature makes it suitable for searches in different brain regions and species. We have annotated all the collected data using a consistent cell-type identification pipeline, enabling comparison of cell types across datasets. Using the cell types identified in our database allows for comparison of similarities and differences between datasets without the need for integration. Our database facilitates exploration of the number of cell types in the brain of each species at the transcriptome level and standardization of cell-type naming. Moreover, integration of genetic markers and other phenotype characteristics for different brain regions of multiple species enables sophisticated research from a comparative perspective. *sc*BrainMap will be updated annually with more collections of cell types, species, disease states and additional phenotypes.

## Supplementary Material

baad035_Supp

## Data Availability

The data underlying this article are available at https://scbrainmap.sysneuro.net/.
