# Supplementary material for: scBrainMap: a landscape for cell types and associated genetic markers in the brain
Source: Database (Oxford). 2023 May 17;2023:baad035. doi: 10.1093/database/baad035 (PMC10191140; doi:10.1093/database/baad035)
Supplement: baad035_Supp [file baad035_supp.zip › suppl_data/TableS1.docx]

| **Species** | **PMID** |
| --- | --- |
| Homo sapiens | 34616062 |
| Macaca fascicularis | 31835035 |
| Callithrix jacchus | 34616062 |
| Mus musculus | 34616062 |
| Rattus norvegicus | 31835035 |
| Mesocricetus auratus | 31835035 |
| Nannospalax galili | 31835035 |
| Ovis aries | 31835035 |
| Gallus gallus | 31835035 |
| Chelydra serpentina | 29724907 |
| Podarcis muralis | 29724907 |
| Danio rerio | 34824389 |
| Astyanax mexicanus | 34824389 |
| Drosophila melanogaster | 29909982 |
