# Supplementary figures and images for: scBrainMap: a landscape for cell types and associated genetic markers in the brain
Source: Database (Oxford). 2023 May 17;2023:baad035. doi: 10.1093/database/baad035 (PMC10191140; doi:10.1093/database/baad035)

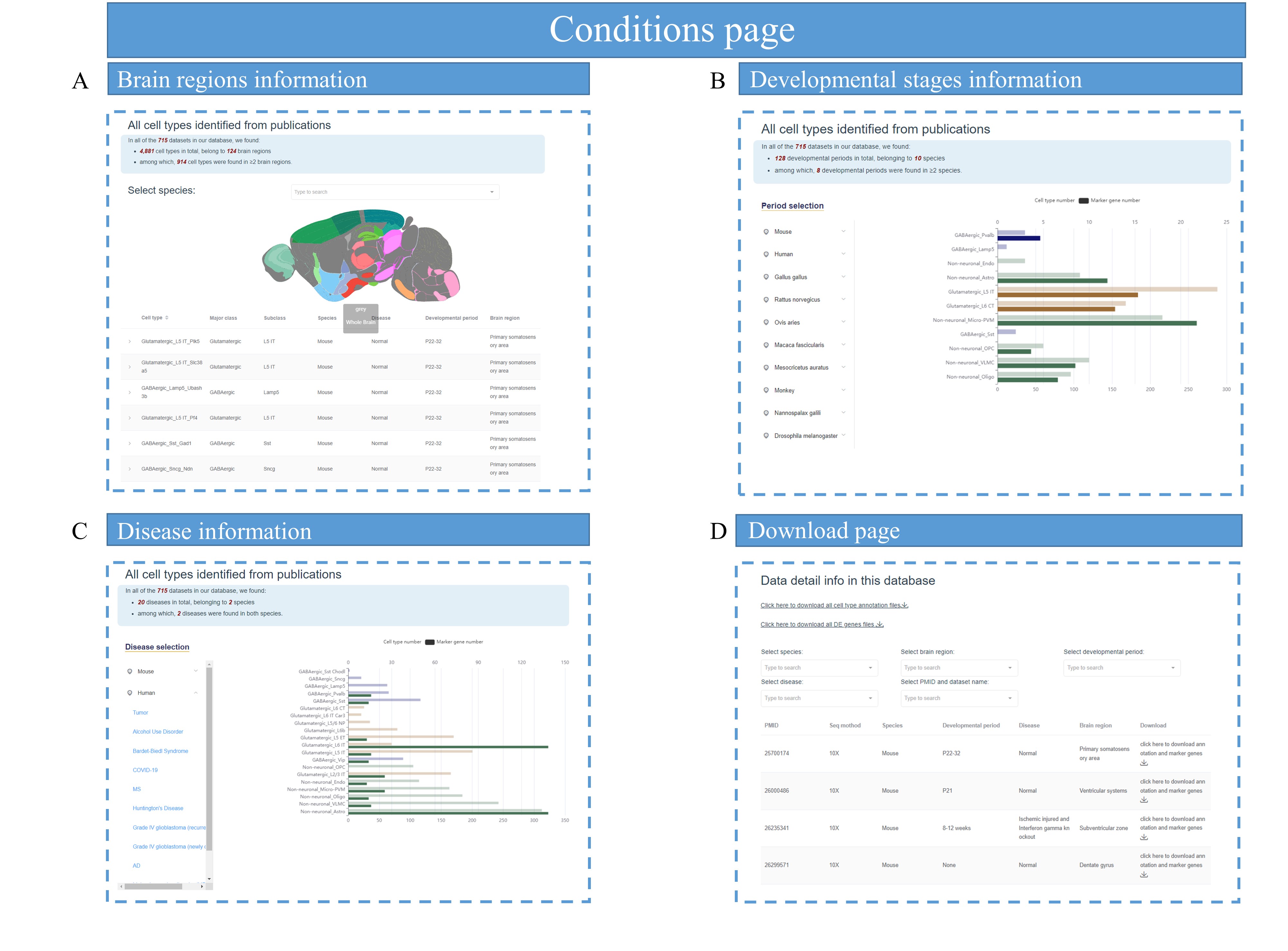

Supplement: baad035_Supp [file baad035_supp.zip › suppl_data/FigureS1.jpg]

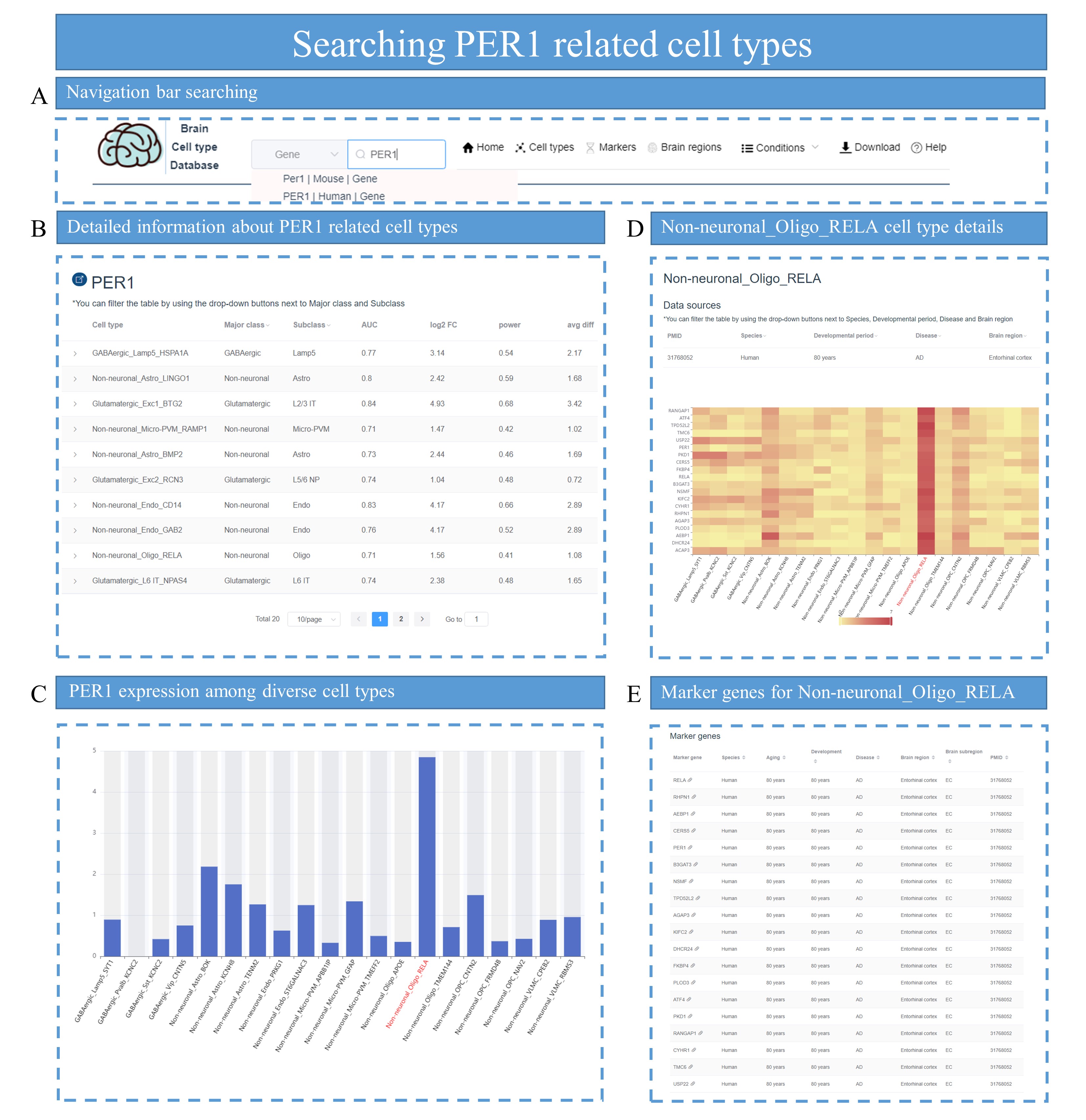

Supplement: baad035_Supp [file baad035_supp.zip › suppl_data/FigureS2.jpg]

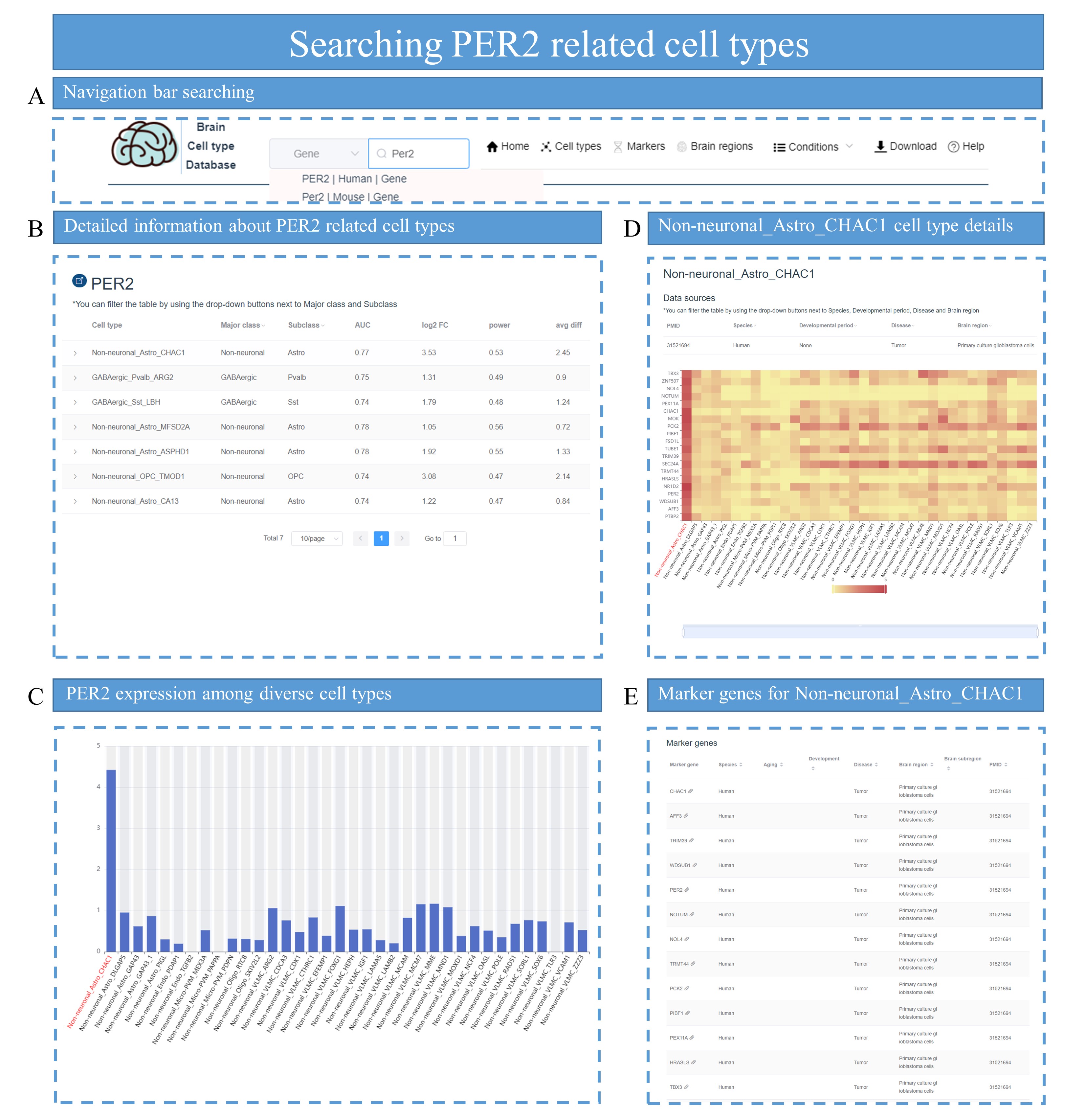

Supplement: baad035_Supp [file baad035_supp.zip › suppl_data/FigureS3.jpg]

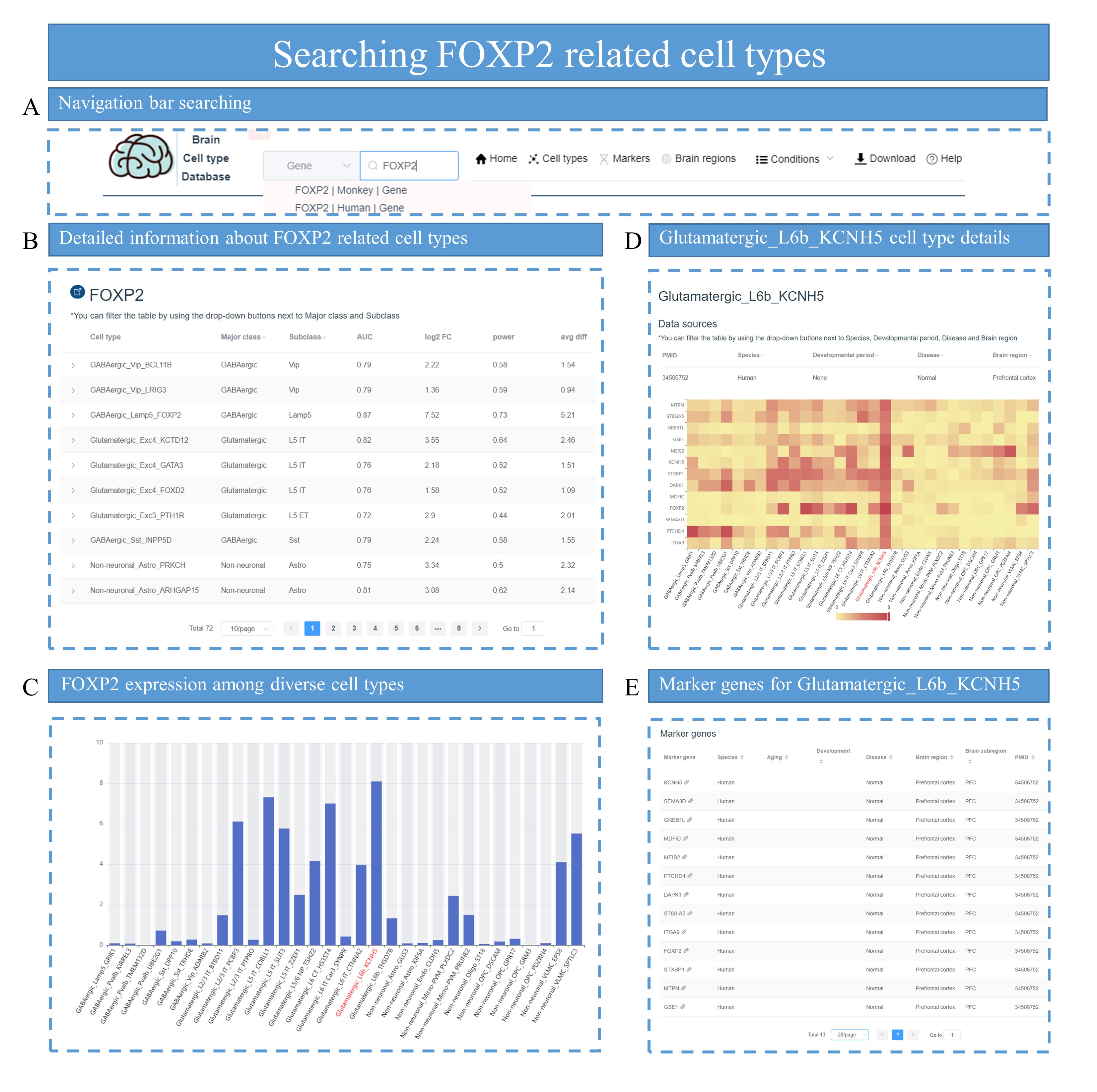

Supplement: baad035_Supp [file baad035_supp.zip › suppl_data/FigureS4.jpg]

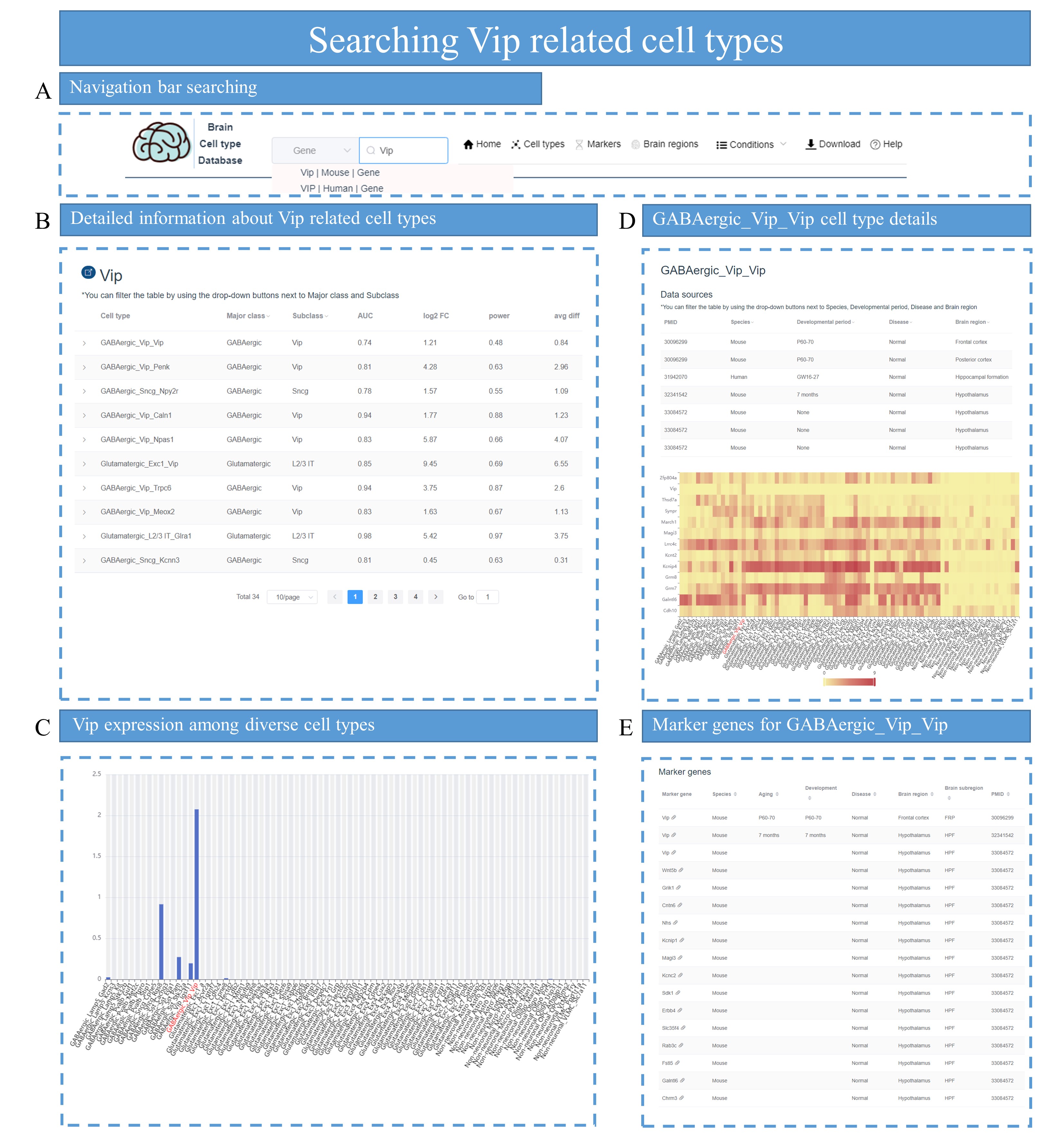

Supplement: baad035_Supp [file baad035_supp.zip › suppl_data/FigureS5.jpg]

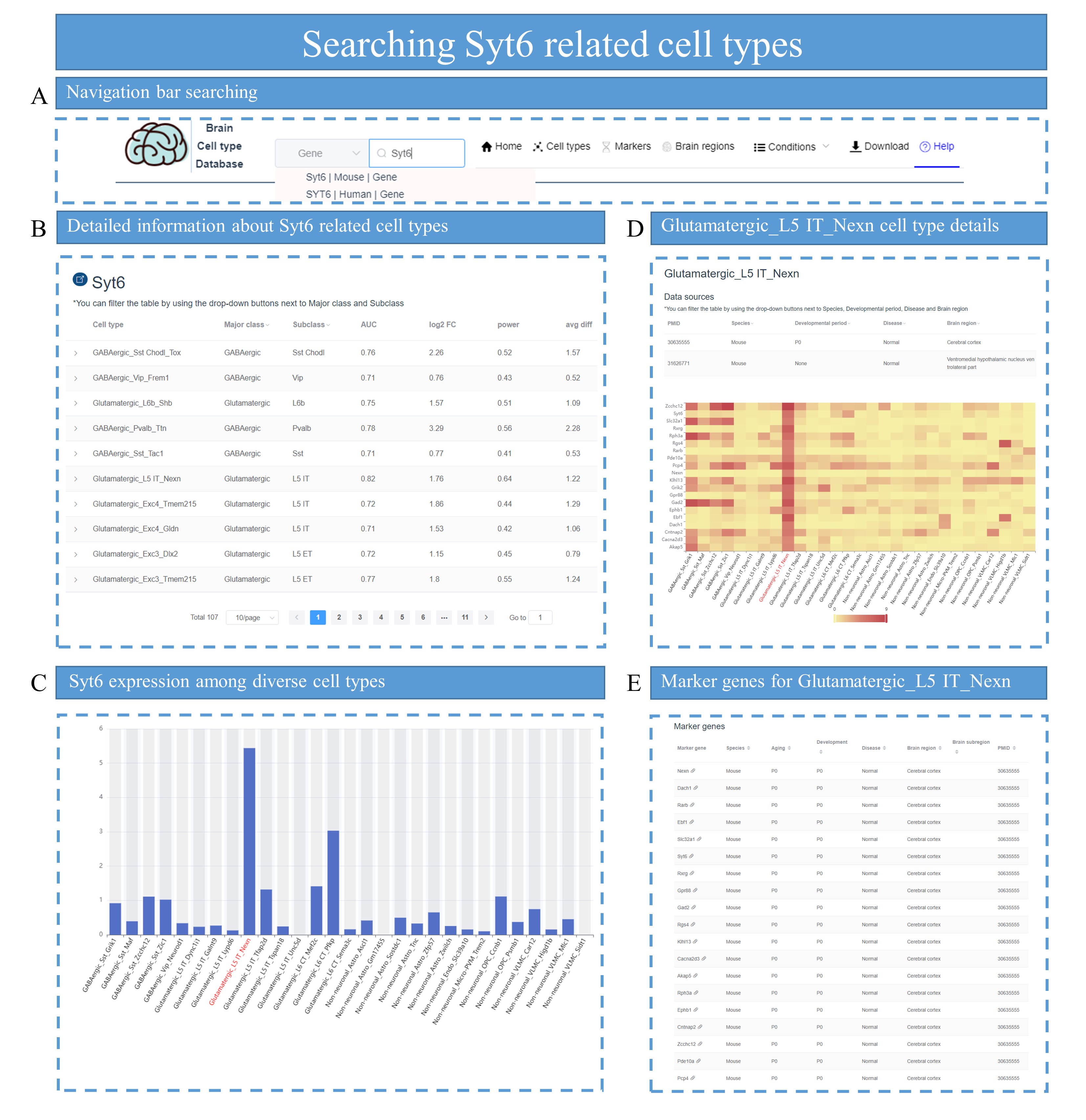

Supplement: baad035_Supp [file baad035_supp.zip › suppl_data/FigureS6.jpg]
